# Stress perception of occupational dermatology patients: a qualitative interview study

**DOI:** 10.3389/fpubh.2026.1844036

**Published:** 2026-06-24

**Authors:** Maurice Waitek, Karoline Lukaschek, Elke Weisshaar

**Affiliations:** Division of Occupational Dermatology, Department of Dermatology, Ruprecht-Karls University Heidelberg, Heidelberg, Germany

**Keywords:** hand eczema, occupational skin disease, psychodermatology, stress, work

## Abstract

**Purpose:**

Patients suffering from a suspected occupational skin disease (OSD) frequently inform their doctors about substantial stressors in their lives. What features characterize their stress however remain ill defined and underresearched. It was aimed at exploring the patients' perspectives on their individual stressors to define relevant outcomes for future patient-centered care studies.

**Materials and methods:**

Qualitative interviews were conducted. Patients with severe hand dermatoses presenting to the division of occupational dermatology were asked 17 open questions on stress that were derived from clinical experience, expert opinions and a preliminary survey of three OSD patients, which were asked about what they felt was important for assessing stress. The results were then processed through qualitative content analysis (summarizing approach).

**Results:**

Sixty-three patients attending a tertiary individual prevention measure were interviewed, 25 were female (39.7%) and the mean age was 49.2 years (SD: 12.0, Min: 21, Max: 63). Interviews took 20 to 45 minutes. Stress was often reported to be job-related (*n* = 28; 44.4%). Patients reported time pressure (*n* = 40; 63.5%) or feeling overworked in uncertain (*n* = 35; 55.6%) and unplannable (*n* = 16; 25.4%) situations as stressful as well as conflicts (*n* = 16; 25.4%) and a perceived transgression of personal and professional limits (*n* = 7; 11.1%). The most frequently reported stress symptoms were irritability (*n* = 32; 50.8%), nervousness (*n* = 19; 30.2%), bodily tension (*n* = 12; 19.0%) and hecticness (*n* = 11; 17.5%).

**Conclusion:**

Job-related stress appears to play an important role in patients with suspected OSD. It is striking that the reported stress symptoms differ from other studies. Based on this interview study, a standardized set of questions to assess stress in OSD will be developed for future research and guide clinicians in what aspects of the stress experience to look out for in their patients.

## Introduction

Occupational skin diseases (OSD), resulting from work in high-risk occupations (e.g., allergic reactions or wet work), affect the global workforce. OSD cohort studies from Finland and Germany have shown that long disease duration, job loss or significant impairment at work are common in spite of therapeutic measures (1, 2). Chronic hand eczema (CHE) is the most common OSD, being defined as persistent signs and symptoms of an inflammatory dermatosis for more than 3 months or with a minimum of two exacerbations over the course of 1 year (3). Patients in Germany, who suffer from severe and chronic hand dermatoses suspected to be OSD, may attend a special rehabilitative measure of tertiary individual prevention (TIP) (4–6). Most patients suffer from CHE, but other hand dermatoses (e.g., palmar psoriasis) are present as well. From 2016 to 2018, 37.3% of these patients were smokers, a higher percentage than in the general population (7). The proportion of patients without vocational training had increased from 11.1% in 2016 to 19.6% in 2018 as well. Low socioeconomic and educational status can act as independent risk factors for the pathogenesis of psychological disorders or psychiatric diseases, inflicting stress on patients (8). During our daily clinical practice we have encountered more frequent reports of these disorders/diseases among our patient population. Patients also regularly reported worries regarding disease progression or possible job loss. However, in the past, these patients often failed to recognize psychosocial factors or even dry skin as potentially important for the formation and maintenance of eczema (9).

Stress as a risk factor plays an increasingly important role in dermatological patients, aggravating the itch-scratch cycle or leading to the exacerbation of skin diseases such as atopic dermatitis (AD) or psoriasis (10–14). Clinical observations and patient reports over the last years revealed an increase in the demand of TIP patients for psychological counseling. These observations suggest that psychosocial factors like stress, critical life events, itch, compliance, or comorbidities such as anxiety disorders and depression can play an important role in the patient's suffering, disease progression and the appropriate care (15). We have previously conducted a systematic literature search on psychosocial aspects of OSD (16). It showed that stress research has been largely neglected in occupational dermatology. Stress was investigated quantitatively in 15 studies mostly reporting cross-sectional and retrospective data. Quantitative measurements of stress establish if patients feel stressed in a given time span, but not how their stress experience manifests itself. CHE Patients in one of the identified studies reported above-average stress levels (17). Another study reported stress possibly hindering healing processes in CHE (18). Similarly, in patients with allergic contact hand dermatitis, reported stress levels were higher than those of healthy controls (19). Recently, exposure to job-related psychosocial stress (e.g., job control, emotional demands, interpersonal conflict) has been positively associated with skin problems (eczema, itchy skin, rash) in both retrospective and prospective analyses (20). No study investigated the stress in OSD patients in a qualitative way. There are, however, qualitative studies on CHE, reporting stress as a trigger for disease flare-ups, alongside recognized irritants such as chemicals and temperature changes (21). Although the CHE patient population overlaps with OSD, the latter are a special patient population needing elucidation with more detailed accounts of their stress experience.

To address the gap of stress research in OSD, the present study explored stress from the patient perspective by conducting a qualitative descriptive study in an exploratory manner. Specifically, we examined characteristics of patient-perceived stress, including how individuals perceived stress (e.g., symptoms), the contexts in which they perceived it (e.g., job-related), and the strategies they employed to cope with it.

## Materials and methods

### Study population

From June 2024 until July 2025, a convenience sample of patients with severe hand dermatoses presenting to the division of occupational dermatology in a TIP measure were approached face-to-face. They were invited to participate in a structured qualitative interview (according to the SRQR Criteria). The aim was to obtain an unbiased open presentation of the patients' views on relevant stressors in their daily lives. Inclusion criteria were admittance to the TIP measure as well as sufficient skills in the German language and informed consent.

### Demographic variables

Demographic variables of patients were assessed, including sex, age, atopy, smoking status (smoker, non-smoker, ex-smoker), occupational group, educational status and disease duration. Occupational groups comprised metal workers, healthcare workers, chemical industry workers, other health-related occupations, food industry workers, construction workers, glass and ceramics industry workers, cleaners and maintenance personnel, plastics industry workers, cosmeticians/podiatrists, retail workers, wood workers, logistics personnel, landscaping/agriculture workers, office workers, printing industry workers, and mining industry workers.

## Structured qualitative interviews

Initially, patients (*N* = 3) were asked in an open format which aspects of stress they considered essential for inclusion in a qualitative stress assessment. These findings were combined with expert input (dermatologist, epidemiologist, psychologist) and clinical experience to develop a list of qualitative-descriptive interview questions assessing relevant stressors (Table 1). This includes stress-specific questions (e.g., regarding coping mechanisms) which are commonly asked during patient counseling at the division of occupational dermatology in Heidelberg. This list was subsequently reviewed for clarity by the participating patients and two independent researchers before being applied to a larger patient sample. A total of 63 patients were then interviewed by a psychologist experienced in occupational dermatology. A standardized interview setting to minimize interviewer and contextual bias was sought. When necessary, questions were clarified to address non-response or misunderstanding. Responses were recorded as field notes directly on the question list, allowing for active paraphrasing and in-interview member checking (e.g., “I will write this down as … Is this what you meant?”).

**Table 1:** Interview questions for qualitative assessement of stress in OSD patients.

Interview questions
1. What does stress mean from your perspective?
2. In what situations do you feel most stressed (work, private life, specific weekdays or people)?
3. Do you enjoy your work?
4. Do you feel appreciated at work?
5. What was the most stressful situation you have ever experienced?
6. How do you notice feeling stressed (physical or psychosocial symptoms)?
7. Do you feel positive stress as well? Something exciting?
8. Do other people tell you when you seem stressed?
9. Would you say that you're stress resistant?
10. How do you deal with conflicts?
11. What do you do to reduce the stress in your life?
12. Did your way of dealing with stress change over time?
13. Are you generally satisfied with yourself?
14. Is it easy to tell the reason you feel stressed?
15. Do you have people you can confide in?
16. Would you say that you are performance-oriented?
17. What do you understand by performance (socially, work-related, free time)?

The questions highlighted in green comprised the first interview questions. Questions 3, 4, 10 and 13 were added later to further investigate discoveries made during the interviewing process and are highlighted in yellow.

During the interviewing process, additional relevant stressors emerged, namely conflicts as a major stressor, a high prevalence of job-related stress and a pronounced performance orientation (i.e., striving to do tasks well and be very productive). These themes arose during the iterative process of data collection and analysis. Consequently, questions 3, 4, 10 and 13 were added at appropriate points in the interview guide to fit the conversational flow. Hence, not all patients answered all questions included in the final version of the interview guide. The percentages reported in the results section therefore refer to the valid responses for each respective question. Missing data resulted both from non-response (e.g., question 5 was occasionally perceived as too personal) and from questions introduced during the ongoing interview process. Results are first presented for the entire study population (*N* = 63), followed by the descriptive statistics restricted to the subsample (*n* = 18) that answered all interview questions.

The qualitative interviews were conducted in the German language and lasted approximately 20–45 min. They were translated into English for the purposes of this publication. Interviews started with the opening statement “I will be conducting this interview with you and ask questions related to stress in your daily life. These questions range from your perception of stress, where stress occurs most often, if you feel any stress symptoms, to your ways of coping with stress and whether you consider yourself performance-oriented or not.” Interviews began with an open question inviting patients to describe what they considered important to convey regarding stress, followed by the structured interview guide (Table 1).

## Data processing and analysis

For data analysis, interview responses were entered into an electronic form and anonymized. Analysis followed the principles of qualitative content analysis (summarizing approach) according to Mayring (22). Data were subsequently processed using descriptive statistical analyses with *IBM SPSS Statistics (version 27)*.

Coding was undertaken by the interviewer (MW) in an iterative process. However, matters were discussed with another author (KL) in cases of unclear admittance to one category. Interviews were analyzed sequentially, with responses from the first interviews used to establish starting categories. Responses from subsequent interviews were then fitted to these categories; if a response did not fit, a new category was established. This process was repeated until no new categories emerged, indicating data saturation in accordance with methods studies (23). As a final quality assurance, all interviews were re-analyzed again using the complete category system.

An interpretivist approach centered on what patients experienced and how they perceived these experiences (what they mean to the patient) was used, reflecting the highly subjective nature of stress and to interpret these experiences in a qualitative-descriptive way with regard to their relevance for the study population. To maintain a focus on recurring themes and patterns, stressors reported by only a single patient were excluded from further analysis, with the exception of stress-related symptoms. These symptoms are inherently subjective and accounted for the largest number of single mentions, in some cases referring directly to cutaneous sensations. A full list of excluded responses is provided in Supplementary Table 1.

Due to the German social security system, informed written consent was provided by consenting to the tertiary individual prevention rehabilitative measure. This includes information necessary for diagnosis and therapy, but is also referring to the psychological sessions and educative measures. Ethical approval has originally been obtained for the measure as a whole in accordance with the Declaration of Helsinki (4–6).

## Results

### Patient characteristics

The mean age of the participants (*N* = 63) was 49.2 years (range: 21–63 years, SD: 12), 25 of which were women (39.7%) and 38 were men (60.3%). Patients were employed in the metal industry (*n* = 18; 28.6%), as healthcare workers (*n* = 11; 17.5%), the chemical industry (*n* = 5; 7.9%), other health-related occupations (*n* = 4; 6.3%), the food industry (*n* = 4; 6.3%), the building sector (*n* = 3; 4.8%), the glass and ceramics industry (*n* = 3; 4.8%), and in cleaning and maintenance (*n* = 3; 4.8%). Further occupational groups were the plastics industry (*n* = 2; 3.2%), as cosmeticians/podiatrists (*n* = 2; 3.2%), the retail sector (*n* = 2; 3.2%), the wood industry (*n* = 1; 1.6%), logistics (*n* = 1; 1.6%), landscaping/agriculture (*n* = 1; 1.6%), office work (*n* = 1; 1.6%), the printing industry (*n* = 1; 1.6%), or the mining industry (*n* = 1; 1.6%). An atopic skin diathesis was present in 39 patients (61.9%). Nineteen patients reported being smokers (30.2%), three were former smokers (4.8%), and 41 were non-smokers (65.1%). The mean disease duration was 12.2 years. 57.1 % (*n* = 36) of patients were married, 20.6% (*n* = 13) were single, 19% (*n* = 12) divorced and 3.2% (*n* = 2) were widowed.

### Stress characteristics

When asked what they felt was important regarding stress, many patients gave no answer, stating they had no special concerns. The patients that did, however, reported conflicting feelings about the definition of stress, stating “never” feeling stressed (Interview 53), stress being “just [their] regular day” (Interview 55) and “things that have never been there before” (Interview 48) being perceived as stressful (Supplementary Table 2).

## Stressful situations

Stressful situations reported by patients were categorized into five groups of stressors (Table 2). Time pressure emerged as the most frequently reported stressor in the overall sample and across most subgroups, ranking second only among smokers and men. In these groups, situational uncertainty/loss of control was most commonly reported. Among patients with atopic conditions and among men, time pressure and situational uncertainty were reported similarly frequently. A lack of future plannability, interpersonal conflicts, and perceived transgression of personal and professional limits were reported much less often.

**Table 2:** Stressors assorted by frequency.

Stressor	Patient reports (*N* = 63)	Men (*n* = 38)	Women (*n* = 25)	Atopics (*n* = 39)	Smokers (*n* = 19)
Time pressure	40 (63.5%)	20 (52.6%)	20 (80%)	25 (64.1%)	9 (47.4%)
Situational uncertainty/loss of control	35 (55.6%)	21 (55.3%)	14 (56%)	25 (64.1%)	11 (57.9%)
Lack of future plannability	16 (25.4%)	8 (21.1%)	8 (32%)	10 (25.6%)	4 (21.1%)
Interpersonal conflicts	16 (25.4%)	10 (26.3%)	6 (24%)	12 (30.8%)	5 (26.3%)
Perceived transgression of personal and professional limits	7 (11.1%)	4 (10.5%)	3 (12%)	4 (10.3%)	2 (10.5%)

Starting categories established after analyzing the first interviews are marked in green. New categories established if an answer did not fit into an existing category are marked in yellow.

The most stressful situations reported were primarily major life events (e.g., loss of a loved one), but also acute stressors such as examinations. Patients stated e.g., the pursuit of their high school diploma without financial support (Interview 42), that their “husband had a cardiac arrest because of the situation with [their] son” (Interview 39), or their father being diagnosed with cancer and wanting to “treat it alternatively first” (Interview 52) (Supplementary Table 2).

## Stress appraisal

Stress was most often perceived in an occupational context (*n* = 28; 44.4%) or in both an occupational and private settings (*n* = 26; 41.3%). Only nine (14.3%) patients primarily attributed their stress to private factors. Patients however reported having fun at work (*n* = 37, 86.0%) and feeling appreciated (*n* = 12, 27.9%) or even very appreciated (*n* = 19, 44.2%). 86.9% (*n* = 53) of our patients reported also experiencing positive stress in their lives.

61.7% (*n* = 37) of patients reported having pointed out to them by others, whenever they seem distressed. In addition, 23.8% (*n* = 15) were not able to tell the origin of perceived stress a lot of the time.

Regarding performance orientation, 82.3% (*n* = 51) of patients reported some degree of performance orientation, while 17.7% (*n* = 11) stated not being performance oriented. Performance orientation was mostly reported in a general context (*n* = 32; 54.2%) or in an occupational context (*n* = 23; 39%). Four patients (6.8%) reported experiencing this exclusively in a private setting.

## Stress symptoms

On average, two stress symptoms were reported per patient. The three most frequently mentioned symptoms were irritability, nervousness and bodily tension, alongside hecticness (Table 3). All patient-reported symptoms are presented in Table 3 in descending order of frequency. Five patients (7.9%) reported no perception of symptoms.

**Table 3:** Stress symptoms experienced by patients.

Stress symptom	Patient reports (*N* = 63)
Irritability	32 (50.8%)
Nervousness	19 (30.2%)
Bodily tension	12 (19.0%)
Hecticness	11 (17.5%)
Exhaustion	10 (15.9%)
Sleep problems	8 (12.7%)
Ruminations	7 (11.1%)
Blushing	6 (9.5%)
Crying	5 (7.9%)
Perspiration	4 (6.3%)
Palpitations	4 (6.3%)
Aching (Nerves, head, stomach)	3 (4.8%)
Nausea/feeling sick	2 (3.2%)
Being quiet	2 (3.2%)
High concentration	2 (3.2%)
Concentration problems	2 (3.2%)
Itch	1 (1.6%)
Blemished skin	1 (1.6%)

Starting categories established after analyzing the first interviews are marked in green. New categories established if an answer did not fit into an existing category are marked in yellow.

## Coping

Concerning strategies to manage stress, most patients reported resting (*n* = 29; 46%), taking walks or going on short trips (*n* = 27; 42.9%) or spending time on hobbies (*n* = 31; 49.2%) and with their families (*n* = 27; 42.9%) as coping strategies. 31.7% (*n* = 20) of patients engaged in sports and 13 patients (20.6%) reported talking about their stress as helpful. Other ways of coping were performing chores in the house and garden (*n* = 9, 14.3%), seeking diversion (*n* = 5, 7.9%) or changing their plans to accommodate the stressor in a better way (*n* = 2, 3.2%). Eating of high calory snacks and smoking as examples of maladaptive coping mechanisms were reported by seven (11.1%) and eight (12.7%) patients, respectively. 84.1% of the interviewed patients felt well supported socially (*n* = 53). Patients took active stances to resolve conflicts (*n* = 27, 67.5%), but also reported conflict avoidance (*n* = 13, 32.5%). They, however, also reported their conflict strategy to depend on the individual situation, conflicts at work or in private, and the amount of contact with the conflict partner. This includes avoidance of the conflict partner “whether at work or in private” with a more likely resolution in private (Interview 56), striving “to solve the conflicts,” but reporting difficulties with conflicts at work (Interview 33), or deciding in the moment whether to “run or stay” (Interview 36) (Supplementary Table 2).

A total of 37.1% of patients (*n* = 23) reported low stress resistance, 48.4% (*n* = 30) described themselves feeling resistant to stress, and 14.5% (*n* = 9) “maybe” felt resistant to stress. They also reported having felt more emotionally stable with growing age (*n* = 38, 65.5%). Those answering mostly felt satisfied with themselves (*n* = 14, 77.8%).

## Final version of the stress interview

The final version of the interview was conducted in 11 male (61.1%) and seven female (38.9%) patients working in the metal industry (*n* = 4, 22.2%), in healthcare (*n* = 5, 27.8%), the chemical industry (*n* = 3, 16.7%), the building sector (*n* = 1, 5.6%), or other occupations (*n* = 5, 27.8%). An atopic skin diathesis was present in eight patients (44.4%). Mean disease duration was 12.8 years. Eight patients reported being smokers (44.4%), two were former smokers (11.1%), and eight were non-smokers (44.4%).

Time pressure was reported by 12 (66.7%) patients, situational uncertainty by nine (50%), conflicts by four (22.2%), a lack of plannability by three (16.7%) and perceived transgression by two patients (11.1%). These were perceived in an occupational or mixed setting by eight patients, respectively (44.4%) and in private by two (11.1%). The perceived stress symptoms (mean: 2.1) ordered by frequency of reports were irritability (*n* = 11, 61.1%), exhaustion (*n* = 5, 27.8%), nervousness (*n* = 3, 16.7%), hecticness (*n* = 3, 16.7%), blushing (*n* = 3, 16.7%), sleep problems (*n* = 2, 11.1%), ruminations (*n* = 2, 11.1%), crying (*n* = 2, 11.1%), perspiration (*n* = 2, 11.1%), bodily tension (*n* = 2, 11.1%), palpitations (*n* = 1, 5.6%), blemished skin (*n* = 1, 5.6%), and concentration problems (*n* = 1, 5.6%).

Coping was performed by simply resting (*n* = 9, 50%) or doing hobbies (*n* = 9, 50%), taking walks or going on short trips (*n* = 7, 38.9%), spending time with family (*n* = 7, 38.9%), sports (*n* = 7, 38.9%), smoking (*n* = 6, 33.3%), doing chores in the house and garden (*n* = 3, 16.7%), eating (*n* = 1, 5.6%) or talking to close persons about it (*n* = 1, 5.6%).

Fun at work was reported by 15 patients (83.3%), and seven patients (38.9%) felt appreciated and six very appreciated at work (33.3%). 77.8% (*n* = 14) experienced positive stress and 77.8% (*n* = 14) also felt resistant to stress as well. Thirteen patients (72.2%) reported having grown more stable over time and 14 patients (77.8%) reported satisfaction with their respective lives and self. Most (*n* = 15, 83.3%) reported good social support.

Twelve patients (66.7%) resolved conflicts actively and six (33.3%) showed avoidant behavior. Eight (47.1%) reported getting spoken to about seeming distressed, with a clear origin of stress in 11 (61.1%) and an unclear one in seven (38.9%) cases.

Twelve patients (66.7%) reported feeling performance oriented, in a work (*n* = 6, 33.3%), private (*n* = 2, 11.1%) or general context (*n* = 10, 55.6%).

## Discussion

Using a qualitative-descriptive approach, this study explored stress and its characteristics from the perspective of patients with severe chronic hand dermatoses suspected of being OSD. Despite evidence suggesting a substantial burden and a growing need for professional mental health support, this patient group has not previously been examined qualitatively with a focus on stress (15, 16, 21).

## Major findings

The most striking finding is that stress was perceived as being primarily of occupational origin. This may be explained by stress in association with occupational environments in general. The occupational position of our patients, often characterized by high job demands and low job control can be classified as stressful (24). Additionally, the burden resulting from impaired hand function and reduced work ability is substantial, given that the hands represent their main “tool” (24–26). In an earlier interview study in patients with refractory OSD of our division (previously named clinical social medicine), these patients reported dejection, helplessness, apathy, insecurity and anxiety about the future as well as feelings of shame and guilt toward the employer (9). They also lacked personal accountability for a more successful disease management. Our patients demonstrated a pronounced (work-related) performance orientation, which may contribute to disregarding symptoms and presenteeism. The aforementioned guilt toward the employer and lack of personal accountability could reinforce this behavior (27). Presenteeism in work-related CHE has been reported compared with a control group, despite reduced work productivity and quality of life (25). Stress at work may grow and subsequently contribute to chronic disease progression in OSD patients (18). Impediment of healing processes can occur as well (18). Job-related stressors have also been shown to increase vulnerability to psychiatric disorders (24). There is an increase in suicide mortality in employees subjected to chronic work stressors (28).

One particularly notable finding concerns the perceived symptoms associated with stressful experiences. These differ from those being assessed by established measurement instruments for somatic symptoms [Screening for somatoform disorders (SOMS) or somatic symptom scale (SSS)] such as e.g., palpitations or gastrointestinal complaints (29, 30). Palpitations were mentioned four times by this patient population, but other symptoms like irritability were much more prevalent in this patient population. Possible reasons for this could be recall bias. Symptoms like irritability can be noticed by others and the patients may be informed that they seem irritable, tense, or exhausted, but other internal sensations are not.

Additionally, for some patients, feelings of burden and stress were reported synonymously, as feelings of burden can be described as stressful, but others differentiated. Question 5 on the most stressful situation ever experienced shows this. Some patients reported solely stressful situations like exams, others reported burdensome events like (impending) deaths of close persons, which they perceived as stressful.

The burden of stress, its' impact and stress awareness cannot be evaluated without considering coping mechanisms. 36.1% of our patients reported feeling a low degree of stress resistance. While not definitively established from this data, insufficient and maladaptive coping mechanisms remain a possible explanation. A qualitative interview study on illness perceptions in OSD patients found mention of psychosocial factors but no patient acknowledgment as a trigger of their skin condition (9). Some, however, acknowledged psychosocial burden and stress to reinforce the disease. A lot of patients thus may not feel the need to successfully cope with stress for the improvement of their disease. Smoking can be classified as maladaptive coping. Nineteen of the interviewed patients reported smoking cigarettes regularly, and eight of them identified smoking as a way of stress reduction in their life. No high-quality evidence links smoking to a higher prevalence of hand eczema (HE) (31). A study in OSD patients participating in a TIP measure however associated smoking with a more severe disease course (32). Smokers had to give up their occupation more frequently than non-smokers (32). Chronic psychosocial distress can lead to inflammation, which can worsen inflammatory skin diseases and initiate symptoms of affective and anxiety disorders (33–37). These symptoms (e.g., fatigue, rumination, sleep loss) may persist due to insufficient/maladaptive coping. In contrast, patient-reported positive stress experiences, feelings of appreciation and joy at work, and self-satisfaction may serve as protective factors, however qualitative data cannot interpret degrees of variance explained for e.g., patient resilience. OSD patient stress resilience should be assessed systematically in future.

## Outlook for future research

The patients presented differing stress perceptions and definitions, influencing their response patterns. This warrants an investigation into more subtle questioning (Question 1) with a broader focus on experiences, sensations and emotions associated with stress.

Questions 7, 10, 12, and 15 on positive stress, conflict management, stress management over time and social support could be omitted in future interviews. The existence of positive stress and a change over time provided little additional information for interpreting patient-reported stress and especially stress chronicity. Question 15 aimed at assessing patient's social support and would be better addressed using a validated quantitative instrument. The answers for question 10 on conflict strategies were inconsistent, with the conflict strategy relying on the situation, the stress levels of the patient and the relationship with the conflict partner. Question 9 on stress resistance resulted in patients often answering that they felt no one is entirely resistant to stress. We therefore suggest rephrasing the question, which may result in more comprehensive and differential answers. Question 14 on being able to tell the origin of stress should be phrased differently as some patients misunderstood the question as being able to speak about stress to others. This was cleared up during the interview process by reiteration of the question by the interviewer, but should be changed in future versions of the interview. Question 6 on stress-related symptoms should be rephrased into a closed question format with the here assigned categories of symptoms to combat the difficulties of free recall.

## Strengths and limitations

The strength of the study is its focus on a specific population of occupational dermatology patients. The open-ended question design allowed for complex, nuanced responses and enabled patients to freely express their views.

However, there are several limitations. The open-ended question approach, while important for exploratory research, in turn limited the level of comparability of the data. A closed-question format may yield more standardized results for future studies. Patients might not be well versed in free recall of stress symptoms in a non-stressful environment, leading to recall bias (especially regarding the stressful situations and symptoms) and impaired reliability. Furthermore, no word-by-word transcript was made, as answers were documented as bullet points during the ongoing interview. In addition, the same person conducted and coded the interviews which might lead to potential bias. However, the established categories were close to the interview content with little room for interpretation for bias reduction. Lastly, the addition of further questions during the interviewing process limits the quantitative interpretability of the patient perception as a whole, but shows that new information can arise and require the adjustment of measurement instruments. The sample size of patients receiving the final version of the qualitative interview was small due to the large number of modifications.

## Conclusion

This study shows that stress is a significant factor in OSD patients, impacting their lives. Stress can be caused by occupational or private factors. Our first results hint to a meaningful contribution of stress felt at work.

Future research should focus on further characterizing stress symptoms in relation to stress perception among OSD patients. Providing a structured list of possible symptoms may overcome the limitations of free recall and yield more comprehensive patient reports. Based on this interview study, a standardized set of questions should be developed for future research. Hence a study is planned to methodically examine this interview on a larger scale.

## Data Availability

The datasets presented in this article are not readily available because this study uses sensitive patient data. Therefore, it cannot be provided to third parties without additional patient consent. Requests to access the datasets should be directed to Maurice Waitek, mauricefrederic.waitek@med.uni-heidelberg.de.

## References

[1] Bentz P Eyerich K Skudlik C Schröder-Kraft C Löffler H Pföhler C et al. Molecular classification in a cohort of occupational dermatological patients: diagnostic results and course of ability to work and sick leave over two years. Acta Derm Venereol. (2026) 106:0023. doi: 10.2340/actadv.v106.adv-2025-0023 41524178 PMC12794301

[2] Mälkönen T Jolanki R Alanko K Luukkonen R Aalto-Korte K Lauerma A . A 6-month follow-up study of 1048 patients diagnosed with an occupational skin disease. Contact Dermat. (2009) 61:261–8. doi: 10.1111/j.1600-0536.2009.01611.x 19878240

[3] Thyssen JP Schuttelaar MLA Alfonso JH Andersen KE Angelova-Fischer I Arents BWM . Guidelines for diagnosis, prevention, and treatment of hand eczema. Contact Dermat. (2022) 86:357–78. doi: 10.1111/cod.14035 34971008

[4] Skudlik C Weisshaar E Scheidt R Elsner P Wulfhorst B Schönfeld M . First results from the multicentre study rehabilitation of occupational skin diseases–optimization and quality assurance of inpatient management (ROQ). Contact Dermat. (2012) 66:140–7. doi: 10.1111/j.1600-0536.2011.01991.x 22070197

[5] Weisshaar E Skudlik C Scheidt R Matterne U Wulfhorst B Schönfeld M . Multicentre study ‘rehabilitation of occupational skin diseases -optimization and quality assurance of inpatient management (ROQ)'-results from 12-month follow-up. Contact Dermat. (2013) 68:169–74. doi: 10.1111/j.1600-0536.2012.02170.x 23046085

[6] Brans R Skudlik C Weisshaar E Scheidt R Ofenloch R Elsner P . Multicentre cohort study ‘rehabilitation of occupational skin diseases—optimization and quality assurance of inpatient management (ROQ)': results from a 3-year follow-up. Contact Dermat (2016) 75:205–12. doi: 10.1111/cod.12614 27356809

[7] Ofenloch R Oesterhelt A Weisshaar E. Rehabilitation program for occupational skin diseases at the University Hospital Heidelberg, Germany: increase in disease duration and age of patients. J Dtsch Dermatol Ges. (2021) 19:746–9. doi: 10.1111/ddg.14444 33932094

[8] Kivimäki M Batty GD Pentti J Shipley MJ Sipilä PN Nyberg ST . Association between socioeconomic status and the development of mental and physical health conditions in adulthood: a multi-cohort study. Lancet Public Health. (2020) 5:e140–9. doi: 10.1016/S2468-2667(19)30248-8 32007134

[9] Bathe A Diepgen TL Matterne U. Subjective illness perceptions in individuals with occupational skin disease: a qualitative investigation. Work. (2012) 43:159–69. doi: 10.3233/WOR-2012-1365 23000640

[10] Lugović-Mihić L Meštrović-Štefekov J Pondeljak N Dasović M Tomljenović-Veselski M Cvitanović H. Psychological stress and atopic dermatitis severity following the covid-19 pandemic and an earthquake. Psychiatr Danub. (2021) 33:393–401. 34795189 10.24869/psyd.2021.393

[11] Hedemann TL Liu X Kang CN Husain MI. Associations between psoriasis and mental illness: an update for clinicians. Gen Hosp Psychiatry. (2022) 75:30–7. doi: 10.1016/j.genhosppsych.2022.01.006 35101785

[12] Birkner T Siegels D Heinrich L Haufe E Abraham S Heratizadeh A . Itch, sleep loss, depressive symptoms, fatigue, and productivity loss in patients with moderate-to-severe atopic dermatitis: analyses of TREATgermany registry data. J Dtsch Dermatol Ges. (2023) 21:1157–1168. doi: 10.1111/ddg.15159 37485573

[13] Cameron S Donnelly A Broderick C Arichi T Bartsch U Dazzan P . Mind and skin: exploring the links between inflammation, sleep disturbance and neurocognitive function in patients with atopic dermatitis. Allergy (2024) 79:26–36. doi: 10.1111/all.15818 37469218

[14] Krstanović K Kroflin K Kroflin L Kušević Z Psoriasis and mental health. A psychodermatological approach with an emphasis on psychotherapy. Psychiatr Danub. (2024) 36:7–16. doi: 10.24869/psyd.2024.7 39546508

[15] Waitek M Weisshaar E. Psychological aspects in occupational dermatology. Dermatol. (2025) 76:57–63. doi: 10.1007/s00105-024-05456-y 39841259

[16] Waitek M Weisshaar E. Psychological cofactors in occupational skin diseases: results of a systematic literature search. Acta DermVenereol (2025) 105:adv44516. doi: 10.2340/actadv.v105.44516 41117226 PMC12676585

[17] Böhm D Gissendanner SS Finkeldey F John SM Werfel T Diepgen TL . Severe occupational hand eczema, job stress and cumulative sickness absence. Occup Med. (2014) 64:509–15. doi: 10.1093/occmed/kqu076 24994848

[18] Olesen CM Agner T Ebbehøj NE Carøe TK. Factors influencing prognosis for occupational hand eczema: new trends. Br J Dermatol. (2019) 181:1280–6. doi: 10.1111/bjd.17870 30851194

[19] Pondeljak N Lugović-Mihić L Davidović BL Karlović D HanŽek M Neuberg M. Serum levels of IL-6 and TNF-α, salivary morning cortisol and intensity of psychological stress in patients with allergic contact hand dermatitis and healthy subjects. Life. (2025) 15:351. doi: 10.3390/life15030351 40141696 PMC11943883

[20] Borge RH Johannessen HA Alfonso JH. Psychosocial work exposures as risk factors for skin problems in a general working population: cross-sectional and prospective associations. Int Arch Occup Environ Health. (2025) 98:309–19. doi: 10.1007/s00420-025-02135-w 40069534 PMC11972187

[21] Grant L Seiding LL Burrows K Belsito DV Weisshaar E Diepgen T . Development of a conceptual model of chronic hand eczema (CHE) based on qualitative interviews with patients and expert dermatologists. Adv Ther. (2020) 37:692–706. doi: 10.1007/s12325-019-01164-5 31956966 PMC7004418

[22] Mayring P. Qualitative Inhaltsanalyse. Grundlagen und Techniken. Weinheim und Basel: Beltz Juventa (2010).

[23] Hennink MM Kaiser BN Marconi VC. Code saturation versus meaning saturation: how many interviews are enough? Qual Health Res. (2017) 27:591–608. doi: 10.1177/1049732316665344 27670770 PMC9359070

[24] Harvey SB Modini M Joyce S Milligan-Saville JS Tan L Mykletun A . Can work make you mentally ill? A systematic meta-review of work-related risk factors for common mental health problems. Occup Environ Med. (2017) 74:301–10. doi: 10.1136/oemed-2016-104015 28108676

[25] Fowler JF Ghosh A Sung J Emani S Chang J Den E . Impact of chronic hand dermatitis on quality of life, work productivity, activity impairment, and medical costs. J Am Acad Dermatol. (2006) 54:448–57. doi: 10.1016/j.jaad.2005.11.1053 16488296

[26] Apfelbacher CJ Ofenloch RF Weisshaar E Molin S Bauer A Mahler V . Chronic hand eczema in Germany: 5-year follow-up data from the CARPE registry. Contact Dermat. (2019) 80:45–53. doi: 10.1111/cod.13113 30246346

[27] Politiek K Oosterhaven JA Vermeulen KM Schuttelaar ML. Systematic review of cost-of-illness studies in hand eczema. Contact Dermat. (2016) 75:67–76. doi: 10.1111/cod.12590 27218305

[28] Baumert J Schneider B Lukaschek K Emeny RT Meisinger C Erazo N . Adverse conditions at the workplace are associated with increased suicide risk. J Psychiatr Res. (2014) 57:90–5. doi: 10.1016/j.jpsychires.2014.06.007 25012186

[29] Rief W Hiller W Heuser W. Screening for Somatoform Disorders (SOMS): development and validation of a screening questionnaire. J Psychosom Res. (2003) 54:339–48.12670611

[30] Gierk B Kohlmann S Kroenke K Spangenberg L Zenger M Brähler E . The somatic symptom scale-8 (SSS-8): a brief measure of somatic symptom burden. JAMA Intern Med. (2014) 174:399–407. doi: 10.1001/jamainternmed.2013.12179 24276929

[31] Loman L Brands MJ Massella Patsea AAL Politiek K Arents BWM Schuttelaar MLA. Lifestyle factors and hand eczema: a systematic review and meta-analysis of observational studies. Contact Dermat. (2022) 87:211–32. doi: 10.1111/cod.14102 35277987 PMC9541324

[32] Brans R Skudlik C Weisshaar E Gediga K Scheidt R Wulfhorst B . Association between tobacco smoking and prognosis of occupational hand eczema: a prospective cohort study. Br J Dermatol (2014) 171:1108–15. doi: 10.1111/bjd.13169 24909920

[33] Boehm D Schmid-Ott G Finkeldey F John SM Dwinger C Werfel T . Anxiety, depression and impaired health-related quality of life in patients with occupational hand eczema. Contact Dermat. (2012) 67:184–92. doi: 10.1111/j.1600-0536.2012.02062.x 22564098

[34] Slavich GM Irwin MR. From stress to inflammation and major depressive disorder: a social signal transduction theory of depression. Psychol Bull. (2014) 140:774–815. doi: 10.1037/a0035302 24417575 PMC4006295

[35] Marron SE Tomas-Aragones L Navarro-Lopez J Gieler U Kupfer J Dalgard FJ . The psychosocial burden of hand eczema: data from a European dermatological multicentre study. Contact Dermat (2018) 78:406–412. doi: 10.1111/cod.12973 29464713

[36] Balieva F Schut C Kupfer J Lien L Misery L Sampogna F . Perceived stress in patients with inflammatory and non-inflammatory skin conditions. An observational controlled study among 255 Norwegian dermatological outpatients. Skin Health Dis. (2022) 2:e162. doi: 10.1002/ski2.162 36479271 PMC9720195

[37] Weisshaar E. Chronic hand eczema. Am J Clin Dermatol. (2024) 25:909–26. doi: 10.1007/s40257-024-00890-z 39300011 PMC11511713

