# Supplementary material for: Stress perception of occupational dermatology patients: a qualitative interview study
Source: Front Public Health. 2026 Jun 24;14:1844036. doi: 10.3389/fpubh.2026.1844036 (PMC13341591; doi:10.3389/fpubh.2026.1844036)
Supplement: Supplementary file 1 [file Table_1.docx]

**Stress perception of occupational dermatology patients: A qualitative interview study**

**Maurice Waitek, Karoline Lukaschek, Elke Weisshaar**

Division of Occupational Dermatology, Department of Dermatology, Ruprecht-Karls University Heidelberg, Heidelberg, Germany

**Journal Name: Frontiers in Public Health, Occupational Health and Safety**

Correspondence:

Maurice Waitek, M.Sc.

Division of occupational dermatology

Department of dermatology

Ruprecht-Karls-University Heidelberg

Voßstraße 2, 69115 Heidelberg, Germany

[MauriceFrederic.Waitek@med.uni-heidelberg.de](mailto:MauriceFrederic.Waitek@med.uni-heidelberg.de)

Supplementary table 1 Stressful situations mentioned only once and thus excluded from further analysis

| Stressful situations mentioned only once |
| --- |
| Incomprehensive leadership at work |
| Daughter is skipping school |
| Being online (and exposed to e.g. phishing) |
| Not feeling needed |
| Applying moisturizers |
| Being around people |
| Making mistakes |
| Initital diabetes diagnosis |
| Everyday life |
| Christmas after the passing of their partner |
| Traveling home to Russia (since the beginning of the war in Ukraine) |
| Energy draining conversations |
| Boredom at work |
| Weltschmerz |

Supplementary table 2 Full patient quotes

| Interview number (patient information) | Quote |
| --- | --- |
| Interview 33  (female, other health-related occupations, aged 29) | “I run or stay, that is decided in the moment” |
| Interview 36  (female, printing industry, aged 27) | “Between friends, I strive to solve the conflicts, but at work it is difficult” |
| Interview 39  (female, retail sector, aged 61) | „My husband had a cardiac arrest because of the situation with our son.“ |
| Interview 42  (female, chemical industry, aged 38) | „Worst was the time, when I caught up on my high school diploma. I had no financial support. I worked saturdays and sundays.“ |
| Interview 48  (male, food industry, aged 57) | „Stress can be unplanned things, but can be planned as well. Simply things that have never been there before.“ |
| Interview 52  (male, landscaping/agriculture, aged 42) | „The worst situation, probably in April, my father was diagnosed with cancer. It’s somewhere between esophagus and stomach, but also on the liver. He wants to treat it alternatively first.“ |
| Interview 53  (male, metal industry, aged 43) | „I basically never have stress, I wouldn’t know the last time I felt stressed.“ |
| Interview 55  (male, metal industry, aged 60) | „I don’t have stress, this is just my regular day.“ |
| Interview 56  (female, other health-related occupations, aged 58) | “I avoid the person, whether at work or in private. There is rarely a resolution, but it is more likely in private.” |
